# Sodium transport system in plant cells

**DOI:** 10.3389/fpls.2013.00410

**Published:** 2013-10-17

**Authors:** Toshio Yamaguchi, Shin Hamamoto, Nobuyuki Uozumi

**Affiliations:** ^1^Department of Microbiology, Faculty of Pharmacy, Niigata University of Pharmacy and Applied Life SciencesNiigata, Japan; ^2^Department of Biomolecular Engineering, Graduate School of Engineering, Tohoku UniversitySendai, Japan

**Keywords:** salt tolerance, Na^+^ transporter, plant, NHX transporters, SOS1 transporters, HKT transporters

## Abstract

Since sodium, Na, is a non-essential element for the plant growth, the molecular mechanism of Na^+^ transport system in plants has remained elusive for the last two decades. The accumulation of Na^+^ in soil through irrigation for sustainable agricultural crop production, particularly in arid land, and by changes in environmental and climate conditions leads to the buildup of toxic level of salts in the soil. Since the latter half of the twentieth century, extensive molecular research has identified several classes of Na^+^ transporters that play major roles in the alleviation of ionic stress by excluding toxic Na^+^ from the cytosol or preventing Na^+^ transport to the photosynthetic organs, and also in osmotic stress by modulating intra/extracellular osmotic balance. In this review, we summarize the current knowledge of three major Na^+^ transporters, namely NHX, SOS1, and HKT transporters, including recently revealed characteristics of these transporters.

## INTRODUCTION

Water availability and its quality greatly affect the growth of most of plant species, and keeping a sufficient amount of water in the soil is of paramount importance for agricultural crop production. Constant irrigation often leads to the accumulation of undesirable salts due to the use of poor quality water, particularly in arid lands, and it has been estimated that about 20% of irrigated land is affected by soil salinity (Yeo, 1999). Salinity stress to most plant species mainly accounts for the increase in cytoplasmic osmotic stress and ion (mainly Na^+^)-specific toxicity (Blumwald, 2000; Blumwald et al., 2000 Munns and Tester, 2008). Overaccumulation of Na^+^ in the cytosol causes inhibitions of protein synthesis, many enzymatic reactions (Hall and Flowers, 1973; Flowers and Läuchli, 1983; Murguía et al., 1995), and photosynthetic processes (Tsugane et al., 1999; Tsunekawa et al., 2009). Exclusion of Na^+^ from photosynthetic organs is therefore crucial for the maintenance of an adequate metabolism and efficient carbon fixation.

**FIGURE 1:**
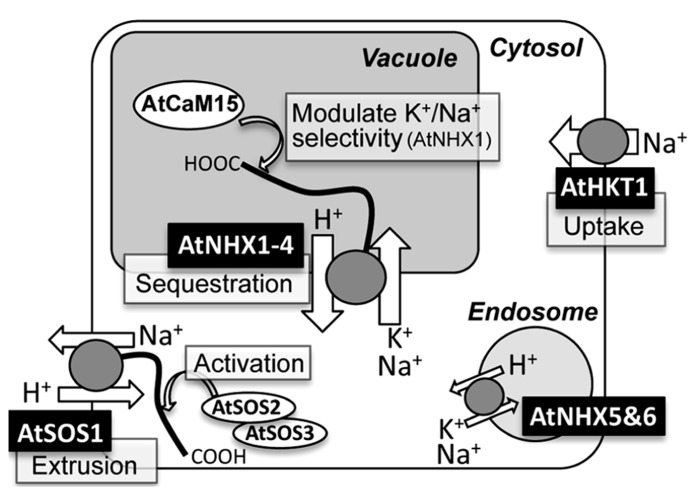
**Schematic representation of subcellular localizations, functions, and regulations of NHX-, SOS1-, and HKT1-type Na^+^ transporters in *Arabidopsis***.

To date, several classes of Na^+^-transporters have been shown to play central roles in Na^+^ homeostasis during salinity stress (Apse and Blumwald, 2007; Hauser and Horie, 2010; Uozumi and Schroeder, 2010). Among them, the NHX-, SOS1-, and the Class I HKT-type transporter have drawn particular attention because of their capability of transporting Na^+^ across cellular membranes (**Figure 1**). Many studies have demonstrated significant involvements of these Na^+^ transporters in Na^+^ sequestration in plant vacuoles, Na^+^ extrusion from cells, and Na^+^ circulation for the alleviation of sodium stress under saline conditions. Moreover, some of these Na^+^ transporters have been shown to improve salinity tolerance of crop plants when their encoding gene is overexpressed (Yamaguchi and Blumwald, 2005; Agarwal et al., 2013).

In this review, we will provide brief summaries on the properties and physiological roles of three major Na^+^ transporters that are involved in the plant Na^+^ tolerance, namely NHX, SOS1, and HKT transporters, from well-established to recently revealed characteristics of these transporters.

## NHX TRANSPORTERS

One of the strategies that plant cells employ for the alleviation of excess cytosolic Na^+^ is to compartmentalize Na^+^ into the vacuoles, and NHX-type intracellular Na^+^/H^+^ exchangers have appeared to be the best-characterized proteins involved in this process so far (Blumwald et al., 2000). Most of NHX proteins studied to date mediate electroneutral Na^+^/H^+^ and K^+^/H^+^ antiport across the membrane by utilizing the H^+^ gradient as a driving force and are considered to be responsible for Na^+^ sequestration into intracellular compartments under salinity stress (Bassil et al., 2012).

Plant genomes often contain multiple intracellular NHX isoforms (Bassil et al., 2012). Intracellular NHX transporters are classified into the IC-NHE/NHX family (Pardo et al., 2006), which is a part of the large cation/proton antiporter 1 (CPA1) family (Säier et al., 2006). These are further subdivided into vacuolar (Class I) or endosomal (Class II) NHXs, according to their sequence similarity and subcellular localizations of representative members (Rodríguez-Rosales et al., 2009; Bassil et al., 2012; **Figure 1**). Most of the plant species sequenced to date contain both types of NHXs (Rodríguez-Rosales et al., 2009; Bassil et al., 2012), and functional redundancies of vacuolar or endosomal NHXs had been recently reported in *Arabidopsis* (Bassil et al., 2011a,b; Barragan et al., 2012).

Currently, two distinct topology models have been proposed for AtNHX1. The first model was proposed based on the protease protection analysis of isolated yeast vacuoles expressing full-length AtNHX1 with epitope tags inserted into each hydrophilic loop (Yamaguchi et al., 2003). This model comprised nine transmembrane segments with a C-terminal hydrophilic tail facing toward the vacuolar lumen. Another topology model was proposed on the basis of *in vitro* translation experiments using truncated *AtNHX1* genes, which suggested that several transmembrane segments of AtNHX1 retain similar topogenic properties as human NHE1 (Sato and Sakaguchi, 2005).

The cation selectivity of AtNHX1 appears to be regulated by its C-terminal tail through the binding to a calmodulin-like protein AtCaM15, which interacts with the C-terminus of AtNHX1 in a Ca^2^^+^ and pH dependent manner from inside of the vacuole (Yamaguchi et al., 2005; **Figure 1**). Interaction with AtCaM15 decreases the Na^+^ transport activity of AtNHX1, while keeping K^+^ transport activity almost unchanged. Given that the plant vacuole usually keeps a high Ca^2^^+^ concentration and an acidic luminal pH, AtNHX1 would favor a K^+^/H^+^ antiport mode under normal physiological conditions due to the binding with AtCaM15. However, under salinity stress, which often causes vacuolar alkalization (Okazaki et al., 1996; Gruwel et al., 2001), disassociation of AtCaM15 would result in increased Na^+^/H^+^ antiport activity of AtNHX1 and promote compartmentation of Na^+^ into the vacuoles (Yamaguchi et al., 2005).

Overexpression of genes encoding vacuolar NHXs has conferred salt tolerance to a range of plant species with a concomitant increase in tissue Na^+^ (Apse et al., 1999; Zhang and Blumwald, 2001; Zhang et al., 2001; Agarwal et al., 2013). These observations, together with the fact that the *Arabidopsis* *atnhx1* mutant exhibited Na^+^ sensitivity and significantly less vacuolar Na^+^/H^+^ antiport activity (Apse et al., 2003), strongly supported the role of vacuolar NHXs in Na^+^ compartmentation under salinity stress. However, there has been a case where the overexpression of vacuolar NHX led to accumulations of K^+^ but not Na^+^ (Leidi et al., 2010). Involvement of endosomal NHXs in salt tolerance has also been demonstrated in tomato and *Arabidopsis*. *LeNHX2* knockdown tomato and *atnhx5*/*atnhx6* double knockout *Arabidopsis* plants exhibited salt sensitivities (Rodríguez-Rosales et al., 2008; Bassil et al., 2011a). The introduction of the AtNHX5 gene increased salt tolerance and Na^+^-accumulation in Torenia (Shi et al., 2008). Tomato plants overexpressing LeNHX2 also showed increased salt tolerance. However, an increase of tissue K^+^ instead of Na^+^ was observed in this case, suggesting that increased salt tolerance was achieved by improving cellular K^+^-homeostasis (Huertas et al., 2013).

Besides significant roles in salt tolerance, involvement in pH regulation has been demonstrated for vacuolar NHXs (Fukada-Tanaka et al., 2000; Yamaguchi et al., 2001). Furthermore, vacuolar NHXs appeared to be involved in organ developments. The *Arabidopsis atnhx1/atnhx2 *double knockout plant exhibited severe defects on the plant growth, development of reproductive organ, and leaf cell expansion under normal conditions (Bassil et al., 2011b; Barragan et al., 2012). These developmental defects seem to be attributed to the role of AtNHXs in K^+^- rather than Na^+^-homeostasis, since the *nhx1-1/nhx2-1* plant accumulated a lower level of K^+^ and exhibited less tonoplast K^+^/H^+^ antiport activity than the wild type plant. Severe growth defects have also been observed in endosomal *NHX* knockout/knockdown plants. The *LeNHX2* knockdown tomato plant showed severely retarded growth as compared to the wild type (Rodríguez-Rosales et al., 2008). Similarly, *Arabidopsis* *atnhx5/atnhx6* double knockout lines exhibited smaller cell size, slower development of flower organs, and root growth (Bassil et al., 2011a). These results may indicate significant contributions of endosomal NHXs on growth and development, possibly via maintaining appropriate pH and/or cation balance in plant cells. Roles in endosomal trafficking have also been documented (Sottosanto et al., 2004; Bassil et al., 2011a), which have been recently summarized in excellent review articles (Bassil et al., 2012; Qiu, 2012) and therefore will not be discussed further.

## SOS1 TRANSPORTERS

Exclusion of Na^+^ from the cytosol across the plasma membrane is also an important mechanism for the alleviation of cellular Na^+^ toxicity in plants (Blumwald, 2000). To date, the SOS1 (salt overly sensitive 1 = AtNHX7 in *Arabidopsis*) transporters are the best-characterized class of transporters attributed with this function (**Figure 1**). The *SOS1* gene has been identified in several plants including *Arabidopsis* (Wu et al., 1996), rice (Martínez-Atienza et al., 2007), wheat (Xu et al., 2008), tomato (Olías et al., 2009), and *Thellungiella salsuginea* (Vera-Estrella et al., 2005). The *SOS1* gene encodes a large membrane protein (127 kDa, in the case of *Arabidopsis* AtSOS1) that is categorized into the CPA1 family along with NHXs, although it is more closely related to bacterial NhaP antiporters (Wu et al., 1996; Shi et al., 2000; Brett et al., 2005). AtSOS1 protein is localized in the plasma membrane, and appears to mediate electroneutral Na^+^/H^+^ antiport (Shi et al., 2002; Qiu et al., 2002,^2003^). Hydropathy prediction has indicated that the AtSOS1 protein consists of 12 putative transmembrane segments and a long C-terminal hydrophilic tail (approximately 700 aa; Shi et al., 2000). Although detailed structural properties of AtSOS1 remain elusive, a recent biochemical analysis revealed that AtSOS1 forms a homodimer (Núñez-Ramírez et al., 2012). In addition, *Arabidopsis* has another *SOS1*-like gene, termed *AtNHX8*, which seems to encode a C-terminal truncated isoform of AtSOS1.**Despite its high similarity to AtSOS1 (~88% for hydrophobic region), AtNHX8 seemed to prefer Li^+^ over Na^+^ as its substrate (An et al., 2007).

It appears that the AtSOS1 C-terminus contains an activation and an auto-inhibitory domain that are physically associated to each other, and this interaction seems to suppress AtSOS1 activity at the resting state (Quintero et al., 2011). The activation of AtSOS1 occurs via the direct phosphorylation of the auto-inhibitory domain by the serine/threonine kinase AtSOS2, which requires a calcium binding protein, AtSOS3, or SCaBP8/CBL10, for its kinase activity (**Figure 1**; Qiu et al., 2002; Quan et al., 2007; Quintero et al., 2011). In addition, AtSOS2 and AtSOS3 have also shown to be required for the Na^+^-dependent induction of *AtSOS1* gene (Shi et al., 2000).

The physiological significance of SOS1 transporters in saline environments has been well established in *Arabidopsis*, tomato, and *T*.* salsuginea*, as significant increases in salt sensitivity has been observed for the knockout/knockdown plants of corresponding *SOS1* genes**(Wu et al., 1996; Shi et al., 2000, 2002; Oh et al., 2009; Olías et al., 2009). All of these knockout/knockdown plants tend to accumulate higher amounts of Na^+^ than wild type plants under severe salt stress, revealing critical roles of SOS1 transporters in limiting Na^+^ entry by promoting Na^+^ efflux at the plasma membrane of the root cells (Shi et al., 2002; Oh et al., 2009; Olías et al., 2009). This is well in agreement with the observation that the *AtSOS1* transcript is predominantly expressed in epidermal cells of the root tip (Shi et al., 2002), and that the overexpression of *AtSOS1*, * T*.* salsuginea* *SOS1*, and rice *OsSOS1* in *Arabidopsis* successfully conferred resistance to salinity stress (Shi et al., 2003; Martínez-Atienza et al., 2007; Oh et al., 2009; Yang et al., 2009). Furthermore, SOS1 appears to play a role in protecting the root plasma membrane K^+^ uptake under salinity stress (Qi and Spalding, 2004). Involvement in xylem loading of Na^+^ for root to shoot transport has also been suggested for AtSOS1 under moderate salt stress and low transpiration conditions (Ding and Zhu, 1997; Shi et al., 2002). This is based on the preferential expression of *AtSOS1* in xylem parenchyma cells and decreased shoot Na^+^ contents in the *atsos1* mutant than in wild type under hydroponic or high humidity conditions (Shi et al., 2002). On the other hand, *SlSOS1*-silenced tomato (*Solanum lycopersicum*) exhibited increased Na^+^ content in roots and leaves as compared to wild type under salt stress, while stems of silenced plants accumulated less Na^+^ than the wild type (Olías et al., 2009). These results prompted a suggestion that SlSOS1 possibly functioned in Na^+^ extrusion from the leaf cells back to the xylem stream in order to protect photosynthetic organs (Olías et al., 2009). SOS1 also appears to have several other cellular functions that are not directly related to Na^+^-homeostasis. A recent study using fluorescent probes reported that the loss of the functional *AtSOS1* gene resulted in altered pH homeostasis, both in the cytosol and the vacuole of root cells, probably due to the alteration in plasma membrane H^+^ flux (Shabala et al., 2005; Oh et al., 2010). In addition, the fragmentation of vacuoles and deficiencies in endocytosis have also been reported in *atsos1* mutants under salinity stress (Oh et al., 2010).

## HKT TRANSPORTERS

The gene encoding high-affinity K^+^ transporter, HKT1 was first identified in the wheat genome by functional complementation screening for genes that rescue the deficiency of TRK1 and TRK2-mediated K^+^ uptake activity in *S*.* cerevisiae* mutants (Schachtman and Schroeder, 1994). HKT1 belongs to the Trk/Ktr/HKT-type K^+^ transporter superfamily found in microorganisms, plant cells, and parasites (Gaber et al., 1988; Ko and Gaber, 1988; Dosch et al., 1991; Schlösser et al., 1991; Schachtman and Schroeder, 1994; Nakamura et al., 1998; Mosimann et al., 2010). The further study on HKT1 revealed that the wheat HKT1 (TaHKT2;1) showed Na^+^/K^+^ co-transport activity at high Na^+^ as compared to K^+^ in extracellular compartment (Rubio et al., 1995; Gassmann et al., 1996). The Na^+^-dependent activation of K^+^ uptake was later found in KtrAB in *Vibrio* *alginolyticus* (Nakamura et al., 1998), and KtrABE in cyanobacteria *Synechosytis* PCC 6803 as well (Matsuda et al., 2004). TbHKT1 in *Trypanosoma* *brucei* (Mosimann et al., 2010) and TsHKT1;2 in *T. salsuginea* (Ali et al., 2012) showed higher selectivity for K^+^ over Na^+^. On the other hand, a strong Na^+^ selective transport activity was found in AtHKT1;1 isolated from *Arabidopsis *(Uozumi et al., 2000). Na^+^ selectivity was also seen in HKTs from other species including *Eucalyptus camaldulensis *and rice (Fairbairn et al., 2000; Horie et al., 2001).

An array of biochemical approaches including bacterial alkaline phosphatase-fusion analysis, glycosylation scanning, and immunofluorescence detection, revealed that AtHKT1 contains eight transmembrane domains that correspond to fourfold bacterial simple K^+^ channels consisting of transmembrane-pore-transmembrane motifs, and N- and C-termini that face the cytoplasm (Kato et al., 2001). Trk/Ktr/HKT-type transporters appear to have evolutional correlation with the ion-conducting regions of K^+^ channels, Na^+^ channels, Ca^2^^+^ channels, and Kdp-type K^+^ pumps**(Durell et al., 1999; Durell and Guy, 1999; Uozumi and Dreyer, 2012). The glycine of the K^+^ ion selective filter in the four pore domains of Trk/Ktr/HKT-type transporters is analogous to the first glycine in the GYG signature sequence (Mäser et al., 2002b; Tholema et al., 2005; Uozumi and Dreyer, 2012). In AtHKT1, the serine in the first pore region in AtHKT1 is substituted for glycine, while the following three glycine residues are conserved. Replacement of the serine by glycine in AtHKT1 led to the increase of K^+^ transport activity, which strongly suggested that AtHKT1 functions as a Na^+^ selective transporter rather than a K^+^ transport system. Based on the relationship of Gly/Ser and Na^+^/K^+^ selectivity in Trk/Ktr/HKT-type transporters in combination with sequence alignments, HKTs were categorized into two major groups (Platten et al., 2006). The conserved arginine in the middle of the last transmembrane domain of Trk/Ktr/HKT-type transporters, which is not conserved in K^+^ channels, is one of the determinants of “transporter” activity but not “channel” activity (Kato et al., 2007). The gating of the ion transport is controlled by the sixth transmembrane in KtrB of *V. alginolyticus* (Hänelt et al., 2010a,b). This structure–function relationship is supported by the crystal structure of a TrkH from *Vibrio* *parahaemolyticus* (Cao et al., 2011).

A long-term exposure of a plant to Na^+^ in soil will translocate Na^+^ from the root to above ground tissue (Mäser et al., 2002a). AtHKT1 may participate in the Na^+^ uptake and Na^+^ circulation in plant body. The analysis of *AtHKT1* gene expression by the use of the GUS reporter system showed that AtHKT1 was expressed in vascular tubes (Mäser et al., 2002a; Berthomieu et al., 2003). This detailed observation, in combination with immunoelectron microscopic detection, showed that AtHKT1;1 resides at the plasma membrane of xylem parenchyma cells and phloem tissues (Berthomieu et al., 2003; Sunarpi et al., 2005). Likewise, one of the rice HKTs was present in xylem parenchyma cells (Ren et al., 2005). The *sas2* (*s*odium over-*a*ccumulation in *s*hoots) mutant exhibited increased Na^+^ accumulation in aerial organs and decreased it in roots (Berthomieu et al., 2003). Taken together, the hypothetical model proposed here to explain the physiological role of AtHKT1 is that AtHKT1 works by unloading Na^+^ from shoot xylem sap of *Arabidopsis* in the presence of high salinity and for recirculation of shoot to root (**Figure 1**; Mäser et al., 2002a; Berthomieu et al., 2003; Sunarpi et al., 2005).

Some transcriptional regulatory elements have been identified in the *AtHKT1* promoter. The tandem repeat regions found far from the *AtHKT1* initiation codon (approximately -3.9 to -5.3 kb) were responsible for the expression in roots (Rus et al., 2006). The second-half of the repeat sequence, which was located in the 3′ region of two repeat sequences, acted as enhancer elements for *AtHKT1* expression (Baek et al., 2011). Reduction of the expression in the root by the inactivation of the repeat sequence induced overaccumulation of Na^+^ in the shoot (Rus et al., 2006). In accordance with this result, the engineered root stele-specific *AtHKT1*-overexpressor reduced salt content in the shoot, whereas *AtHKT1* expression driven by the *35S* promoter in whole plant resulted in salt hypersensitivity (Møller et al., 2009). The putative highly methylated sequence (250 bp) was found 2.6 kb upstream from the initiation codon of *AtHKT1*. It is believed that this region is a potential small RNA target site and the methylation in this region may control the expression of *AtHKT1* at a low level (Baek et al., 2011).

## CONCLUDING REMARKS

Almost two decades of extensive molecular studies have clearly established the involvement of NHX, SOS1, and HKT transporters in plant salt tolerance. The genes encoding some of these transporters have since been successfully utilized as genetic tools for enhancing salt tolerance of model and crop plants. Recent advancements have also provided evidence on the involvement of these transporters in other vital cellular processes. This indicates the possibility that further enhancement of salt tolerance by overexpression of any of the above-mentioned genes might reach its limit due to excessive perturbation of related cellular and physiological processes. A much broader and profound understanding of the molecular properties, regulations, and cellular/physiological functions of Na^+^-transporters, may provide further insight into the development of efficient strategies for the engineering of “super salt-tolerant” crop plants and therefore progress in this research field is highly anticipated.

## Conflict of Interest Statement

The authors declare that the research was conducted in the absence of any commercial or financial relationships that could be construed as a potential conflict of interest.
